# Branch retinal artery occlusion time to presentation and diagnosis: a retrospective review

**DOI:** 10.1186/s40942-026-00815-4

**Published:** 2026-02-21

**Authors:** Hannah El-Sabrout, Daniel Lee, Ronak Shah, Aubrey L. Gilbert, Amar Patel, Dana Sax, Mary Reed, Mubarika Alavi, Nikita Vora, Cindy Hwang, Tova Mannis, Adrian Dokey, Shannon Moore, Robin A. Vora

**Affiliations:** 1 School of Medicine, University of California, San Francisco, San Francisco, CA USA; 2 Drexel University College of Medicine, Philadelphia, PA USA; 3 Cleveland Clinic Cole Eye Institute, Cleveland, OH USA; 4 Department of Ophthalmology, Kaiser Permanente Northern California, Vallejo, CA USA; 5 Department of Ophthalmology, Kaiser Permanente Northern California, Oakland, CA USA; 6 Department of Emergency Medicine, Kaiser Permanente Northern California, 275 W. MacArthur Blvd, Oakland, CA 94612 USA; 7 Department of Research, Kaiser Permanente Northern California, Oakland, CA USA; 8 Emory University School of Medicine, Atlanta, GA USA; 9 School of Medicine, The University of New Mexico, Albuquerque, NM USA

**Keywords:** Branch retinal artery occlusion, BRAO, Retinal vascular disease

## Abstract

**Background:**

Branch retinal artery occlusion (BRAO) is an acute, vision-threatening condition that often signals underlying systemic vascular disease and the need for urgent vascular risk assessment and mitigation. Although prompt evaluation is critical for accurate diagnosis and early identification of modifiable vascular risk factors, patterns of clinical presentation remain poorly characterized.

**Methods:**

This retrospective study included BRAO patients who presented to Kaiser Permanente Northern California (KPNC) within 30 days of symptom onset from 2014 to 2023. Demographic data, symptom timing, healthcare contact, and ophthalmologic evaluation were collected. The type of contact was categorized as eye care provider, call center, emergency department/urgent care, or other. Delays in presentation were defined as time from symptoms to initial contact and were analyzed across subgroups.

**Results:**

From 2014 to 2023, 760 patients were diagnosed with acute BRAO. Mean age of the study population was 70.2 ± 12.6 years, and 330 (43.4%) were female. Initial contact most commonly occurred with eye care providers (370; 48.7%), followed by call centers (251; 33.0%), emergency/urgent care (73; 9.6%), and other providers (66; 8.7%). Only 219 (28.8%) presented within 1 day of symptom onset, while 97 (12.8%) presented after 8 days. The majority (541; 71.2%) were not evaluated by an eye care provider until > 24 h after symptom onset. Among the 153 (20.1%) with known time of symptom onset, median delay to any healthcare contact was 4.0 h. 80 (52.3%) patients presented within 4.5 h, but just 5 (6.3%) were seen by an eye care provider within that window.

**Conclusions:**

In a large, multi-center, community population, delays in BRAO care were common. Although no Level 1 evidence currently exists to support BRAO treatment, success of future therapies will likely require early administration. Prompt evaluation is also critical to enable risk assessment and mitigation. Most patients in this cohort presented in a delayed fashion, underscoring the need for public education and workflow improvements to support earlier recognition and care.

## Précis/Summary statement

Patients with branch retinal artery occlusion often experience delays in care. Most are not evaluated promptly by eye care providers, which may preclude timely diagnosis, stroke prevention, and potential future therapies.

## Background

Branch retinal artery occlusion (BRAO) is an acute retinal vascular event that results in sudden, painless monocular visual loss affecting a portion of the visual field [1, 2]. Although central retinal artery occlusion (CRAO) has historically received more attention, BRAO can also lead to lasting visual impairment and diminished quality of life [1–4]. The natural history of BRAO is often more favorable than CRAO, but meaningful visual recovery is not certain [1, 2, 5]. Additionally, like CRAO, BRAO is associated with heightened risk of other serious vascular events such as cerebral stroke, particularly around the time of occurrence [6–9]. 

Emerging evidence suggests that systemic thrombolysis may offer visual benefit in CRAO when administered promptly, and ongoing research continues to explore its utility [10–14]. More recently, a systematic review evaluating thrombolysis specifically in patients with BRAO reported encouraging visual outcomes, identifying both time to presentation and baseline visual acuity as independent predictors of final vision [15]. Given the shared pathophysiology, it is conceivable that BRAO may respond to early thrombolytic intervention [14, 16], further strengthening the rationale for emphasizing time-critical recognition and triage, although no high-quality studies have evaluated this to date. Apart from consideration of visual recovery, in light of the risk of associated vascular events, current American Heart Association and American Stroke Association guidelines both recommend urgent neurovascular evaluation for all patients with acute retinal artery occlusion, regardless of type [17, 18]. 

Prior work has demonstrated that delays in care are common among CRAO patients, often rendering them ineligible for time-sensitive interventions and delaying appropriate vascular risk mitigation [11, 19]. In contrast, the presentation patterns of patients with BRAO remain poorly characterized, despite emerging evidence that time to presentation may influence outcomes in BRAO. To address this gap, we performed a retrospective cohort study across a large, diverse, integrated healthcare system to characterize presentation timelines and triage pathways in patients diagnosed with acute BRAO. Understanding how patients access care is a critical first step toward designing public education efforts and improving system-level infrastructure to support timely diagnosis, vascular risk mitigation, and any potential future time-sensitive therapeutic interventions.

## Methods

Kaiser Permanente Northern California (KPNC) is an integrated healthcare delivery and insurance system serving over 4.5 million members. Members of KPNC include approximately 33% of the population in areas served and are representative of the demographic and socioeconomic diversity of the surrounding and statewide population [20, 21]. We used International Statistical Classification of Diseases, Ninth and Tenth Revision (ICD-9 and ICD-10) [22] to identify all patients with a diagnosis of branch retinal artery occlusion (BRAO) between October 1, 2014, and December 31, 2023.

A total of 1,410 patient charts were initially retrieved. Each chart, along with associated multimodal retinal imaging, was independently reviewed by fellowship-trained retina specialists (RV, AP, CH) to confirm the diagnosis. Diagnostic confirmation required clinical documentation of an acute, sectoral retinal ischemic event consistent with BRAO, supported by multimodal imaging when available, including color fundus photography demonstrating sectoral retinal whitening, optical coherence tomography (OCT) showing inner retinal hyperreflectivity and edema, and/or fluorescein angiography revealing delayed or absent arterial filling in the affected arterial branch. Patients were excluded if the diagnosis of BRAO was not supported by clinical documentation or imaging, or if findings were more consistent with isolated cotton wool spots or paracentral acute middle maculopathy (PAMM) without other characteristic signs of BRAO. Furthermore, cases of isolated cilioretinal artery occlusion were excluded, as were patients with BRAO due to non-embolic etiologies. Finally, patients with a remote history of BRAO or with non-acute presentation -- defined as evaluation > 30 days after symptom onset -- were excluded. After applying these criteria, 760 patients with confirmed acute ischemic BRAO were included in the final analytic cohort.

In patients presenting later in the disease course, when retinal edema may have partially or completely resolved, diagnosis was based on documented acute symptom onset in combination with residual structural OCT changes and/or corroborating examination and imaging findings consistent with prior branch retinal ischemia. Advanced functional testing modalities, such as microperimetry, were not routinely available and therefore were not required for diagnostic confirmation.

Data extracted included demographic variables (age, sex, race), and clinical timeline variables: date and time of symptom onset, first healthcare system contact, ophthalmologic evaluation, and diagnosis (reviewed by: HES, DL, NV, SM). The first point of contact was categorized into one of four settings: (1) eye care provider, (2) call center, (3) urgent care/emergency department, or (4) other (e.g., neurology, primary care provider). Symptom onset time was determined through review of all relevant clinical notes, with discrepancies adjudicated by giving priority to the ophthalmologist’s recorded history. Time of first healthcare contact and time of ophthalmologic evaluation were defined using the creation timestamps of the relevant progress notes in the medical record.

Due to inconsistent documentation of exact symptom onset time in a subset of patients, the primary analysis used calendar date-based intervals to categorize delays in presentation: <1 day (0–23 h), 1–3 days, 4–7 days, and > 8 days. For patients with a clearly documented time of symptom onset, a secondary analysis was conducted to assess mean time to presentation and proportion of patients presenting within 4.5 h.

This retrospective study was approved by the KPNC Institutional Review Board and conducted in accordance with the Declaration of Helsinki. Informed consent was not obtained as only de-identified data were analyzed. Statistical analysis was performed using Microsoft Excel (Redmond, Washington, USA) and R, version 4.4.0 (Vienna, Austria). Quantitative and descriptive statistics were used to characterize presentation delays, stratified by race and type of initial healthcare contact. Mean delays were visualized using bar graphs, and differences in presentation time across racial groups were assessed using one-way analysis of variance (ANOVA). Race/ethnicity categories reflect the terms used in the electronic medical record, which may be patient or provider ascribed. Medians were used in instances with significant outliers, leading to skew of the mean.

## Results

From October 1, 2014, to December 31, 2023, 760 patients were confirmed to have acute branch retinal artery occlusion (BRAO) and documented presentation to the healthcare system within 30 days of symptom onset. Of the 760 patients, 430 (56.6%) were male and 330 (43.4%) were female, with a mean age at symptom onset of 70.2 ± 12.6 years. The race/ethnicity distribution included 526 (69.2%) Non-Hispanic White, 85 (11.2%) Asian, 37 (4.9%) Black, 82 (10.8%) Hispanic, and 30 (4.0%) patients of other racial backgrounds, including American Indian or Alaska Native, Native Hawaiian or other Pacific Islander, multiple races or ethnicities, unknown or missing. BRAO involved the right eye in 441 patients (58.0%), the left eye in 316 (41.6%), and was bilateral in 3 (0.4%). Initial visual acuity at presentation was frequently preserved: 65.3% (496/760) presented with 20/40 or better, 25.8% (196/760) presented between 20/50 and 20/200, and 9.00% (68/760) presented with worse than 20/200.

**Fig. 1:**
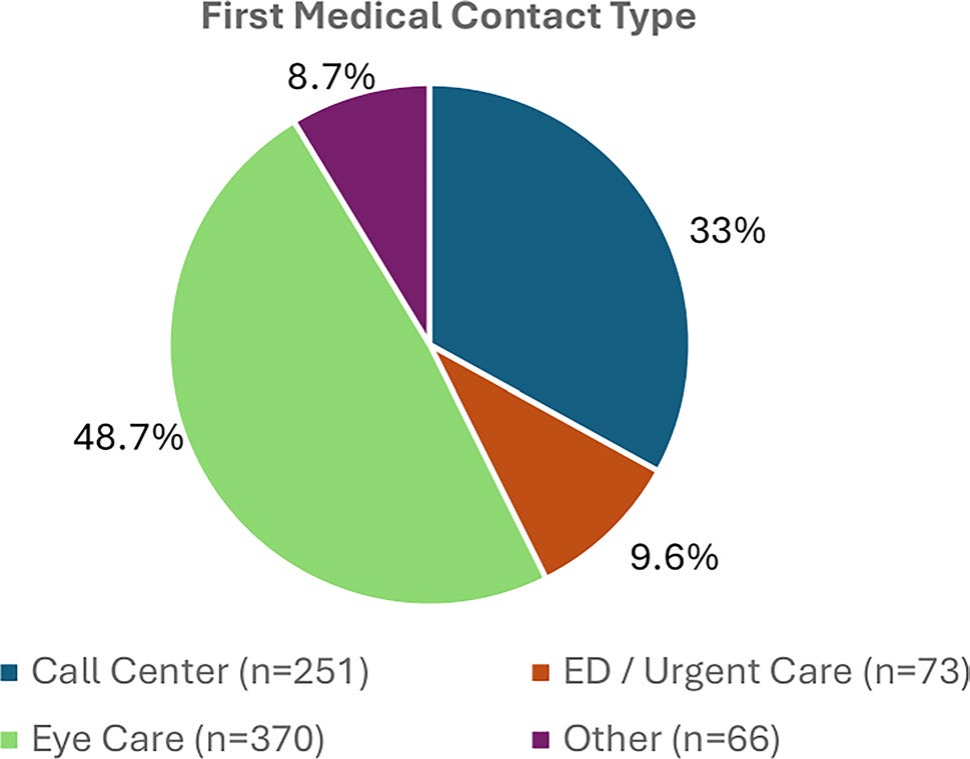
Initial medical contact across the study population

There was substantial variability in the type of healthcare provider or venue first contacted. Among the 760 patients, initial contact occurred most commonly with an eye care provider (ophthalmologist or optometrist) followed by a call center, the emergency department or urgent care, and other types of providers, respectively (Fig. 1). Of those who first contacted the call center (251; 33.0%), 183 (72.9%) were seen next by an eye care provider, and 68 (27.1%) were first directed to emergency care. Among patients whose initial contact was categorized as “other” (66; 8.7%), 62 (94.0%) were later evaluated by an eye care provider.

**Fig. 2:**
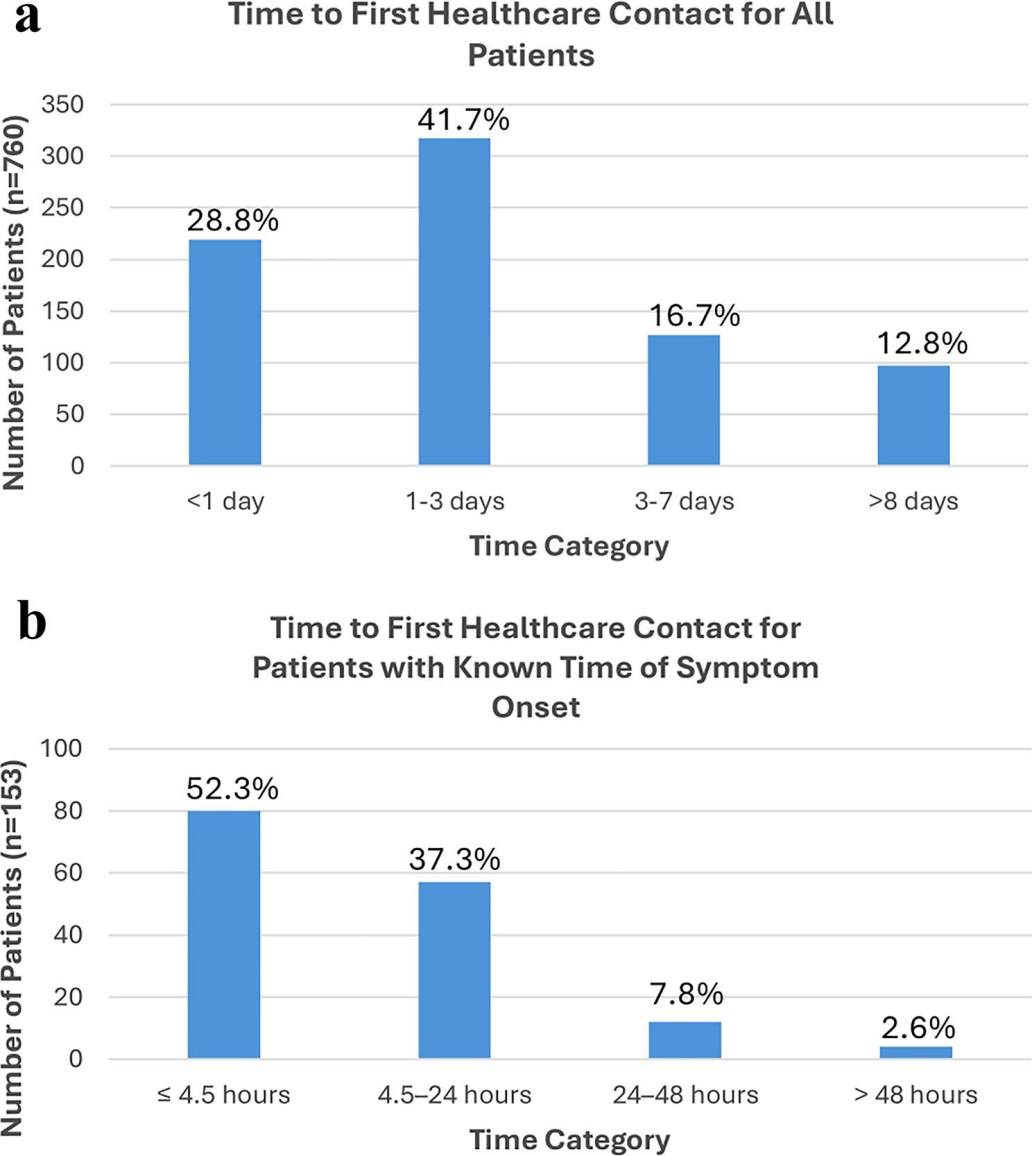
**a**. Time from symptom onset to patient presentation. **b**. Time from symptom onset to patient presentation for the subset of patients with known exact time of symptom onset

Among all 760 patients, fewer than one-third (28.8%) presented within 1 day of symptom onset, while the majority presented after the first 24 h (Fig. 2a). 317 (41.7%) presented between 1 and 3 days, 127 (16.7%) presented between 3 and 7 days, and 97 (12.8%) presented after 8 days. Mean time to healthcare contact varied by racial group: Hispanic (4.1 ± 5.3 days), White (3.2 ± 4.7 days), Asian (3.8 ± 4.9 days), Black (3.6 ± 4.8 days), and other (3.7 ± 4.5 days); however, the differences in mean time to healthcare contact were not statistically significant (*p* = 0.98). Delays in presentation were common across all racial and ethnic groups. Although Hispanic patients had the numerically longest mean delay, prolonged time to presentation was observed across all racial and ethnic groups, suggesting that delayed presentation is a general phenomenon rather than isolated to a specific population.

The average times to patient presentation from October 2014 to May 2019 (i.e., the first half of the study) compared with June 2019 to December 2023 remained similar (3.6 ± 4.8 days versus 3.2 ± 4.8 days, respectively). Overall, 460 patients (59.3%) did not receive ophthalmologic evaluation until more than 24 h after symptom onset. The median time from symptom onset to ophthalmologic evaluation across the cohort of patients who saw an eye care provider at some time point was 3.5 days.

One hundred fifty-three patients had clear documentation of exact time of symptom onset, and in this subgroup, the median time to first healthcare contact was 4.0 h (range 11.6). A total of 80 patients (52.3%) presented within 4.5 h of symptom onset, while the remainder presented beyond this window (Fig. 2b). Among the 80 patients who presented to the healthcare system within 4.5 h, only 5 (6.3%) presented directly to an eye care specialist.

Fundus photography availability varied: 20.8% (158/760) had fundus imaging within 30 days of presentation, 22.5% (171/760) within 90 days, and 40.0% (304/760) had fundus imaging available at any time following the retinal artery occlusion event.

## Discussion

In this large, multicenter cohort study of 760 patients with acute branch retinal artery occlusion (BRAO), we identified notable variation in time to presentation, first contact with a medical specialist after symptom onset, and definitive diagnosis by an ophthalmologist. Only 34.3% of patients presented within 1 day of symptom onset, and over half (59.3%) of all patients were not seen by an eye care provider for more than 24 h. Of the patients who had a documented time of symptom onset, almost half (45.5%) presented after 4.5 h, a time window that has been identified as clinically relevant in emerging studies of retinal ischemia.

These findings are broadly consistent with prior literature on central retinal artery occlusion (CRAO), for which delayed presentation is also common [23–28]. However, our findings suggest that delays may be even more pronounced in BRAO. Compared to CRAO, patients with BRAO presented later, were less likely to seek care through emergency departments, and more often initiated contact through non-urgent pathways such as call centers [29]. This difference is intuitive, as CRAO typically results in profound, complete vision loss, whereas BRAO often causes partial field deficits that may be underappreciated by patients. Nevertheless, recent evidence suggests that time to presentation and baseline visual acuity may influence visual outcomes in BRAO,^15^ reinforcing the importance of early recognition.

Emerging evidence underscores the critical importance of prompt systemic evaluation following RAO [30]. A Korean nationwide study found that patients with CRAO had a significantly elevated risk of ischemic stroke, particularly within the first week after the occlusion [31]. Similarly, a large U.S.-based cohort study utilizing the TriNetX database reported that patients with RAO had a 1.72% risk of stroke within two weeks and a 2.48% risk within 30 days post-RAO, significantly higher than matched controls [32]. These findings highlight the necessity of immediate assessment for underlying risk factors and implementation of stroke prevention strategies in patients presenting with RAO, including BRAO, to mitigate the heightened risk of subsequent vascular events.

The longer average time to ophthalmologic evaluation in BRAO relative to CRAO further highlights a lack of perceived urgency surrounding this condition– by both patients and providers. Time to ophthalmologic evaluation is often measured in days or weeks rather than hours. While most patients ultimately received specialist ophthalmic evaluation, the route of initial contact likely influenced the speed of diagnosis and later referral to ophthalmology. To reduce these delays, health systems could deploy nonmydriatic fundus photography [33, 34] or combined fundus photography/OCT devices [35] in frontline settings such as emergency departments and urgent care centers. Artificial intelligence–assisted image interpretation may further accelerate recognition and referral in the future [36]. These tools, coupled with streamlined triage protocols and tele-ophthalmology support, could help alleviate diagnostic bottlenecks. Since definitive stroke referrals are often initiated only after retinal ischemia is recognized, delays in reaching eye care services, such as gaps in ophthalmoscopy proficiency among non-ophthalmologists, may translate into delays in systemic evaluation and stroke risk mitigation. This finding underscores the importance of clinician education across care settings, as well as the implementation of assistive diagnostic technologies and enhanced patient education.

Although no Level 1 evidence currently exists to support thrombolytic therapy for either CRAO or BRAO, recent systematic review data suggest potential benefit of thrombolysis in BRAO, with time to presentation identified as a key predictor of outcome [15]. Any future treatments --whether thrombolytic or otherwise [37]-- will almost certainly be time-sensitive. Our findings indicate that most patients with BRAO would present too late to be eligible for any such potential interventions. Notably, although not statistically significant, there was a trend toward Hispanic patients having the longest mean delays, highlighting disparities that may stem from structural barriers, differences in health literacy, or healthcare access inequities. Rather than indicating disparities affecting only specific populations, this pattern may further reflect a broader lack of public awareness regarding the urgency of sudden visual field changes across demographic groups. These patterns mirror findings in CRAO and broader stroke literature [11, 38–41], underscoring the need for targeted intervention.

Public health education must reinforce that acute monocular vision loss—whether complete or partial—can signal an ischemic stroke. Campaigns such as BE-FAST and the Canadian SUDDENS mnemonic have successfully broadened stroke recognition to include visual symptoms [42, 43]. Ensuring that these messages reach vulnerable populations, particularly disadvantaged racial and ethnic minority groups, is critical [44, 45]. Additionally, enhanced training for call center staff and frontline clinicians is crucial to improve recognition that acute partial vision loss can indicate stroke and requires urgent investigation as well as expedited ophthalmologic referral.

Limitations of this study include its retrospective design and reliance on unstructured documentation. Progress note timestamps were used as a proxy for the times of patient presentation to the healthcare system and to the ophthalmologist, which may not precisely reflect actual encounter times. This approach likely underestimates true delays in presentation, as symptom onset or initial patient contact may precede documentation. Additionally, our findings may be unique to our institution due to its integrated structure, which facilitates rapid communication among staff and physicians across departments and leverages a large network of advice nurses to enable timely triage. Incomplete or inconsistent documentation of symptom onset time and presenting features may have limited precision in subgroup analyses and could have affected comparisons across presentation pathways. Finally, patient identification depended on diagnostic codes entered by physicians, so miscoding may have resulted in missed cases of CRAO during the study period. Similarly, variability in documentation of presenting symptoms and visual acuity limited our ability to quantitatively analyze these features across the full cohort.

## Conclusions

This study adds to the growing body of literature highlighting critical delays in the presentation and diagnosis of retinal arterial occlusions. Delays in both presentation and diagnosis may preclude future therapeutic opportunities and slow stroke prevention efforts. Based on the observed patterns of delayed and frequently non-urgent presentation, targeted public education emphasizing that acute partial visual field loss may represent an ischemic event, clinician education across care settings, and refinement of triage and referral workflows appear to be the most impactful areas for system-wide improvement. Future work should explore interventions to improve recognition, accelerate routing to ophthalmology, and assess how time to diagnosis impacts visual and systemic outcomes.

## Data Availability

The datasets used and/or analysed during the current study are available from the corresponding author on reasonable request.

## Abbreviations

BRAO: Branch Retinal Artery Occlusion
KPNC: Kaiser Permanente Northern California
CRAO: Central Retinal Artery Occlusion
ICD-9: International Statistical Classification of Diseases, Ninth Revision
ICD-10: International Statistical Classification of Diseases, Tenth Revision
PAMM: Paracentral Acute Middle Maculopathy
USA: United States of America
ANOVA: Analysis of Variance
RAO: Retinal Artery Occlusion
OCT: Optical Coherence Tomography
BE-FAST: Balance, Eyes, Face, Arms, Speech, Time (stroke recognition mnemonic)
SUDDENS: Sudden Unilateral Visual Loss, Drooping Face, Difficulty Speaking, Severe Headache (stroke recognition mnemonic)
